# Adventures of Clinical Psychology

**DOI:** 10.3390/jcm10214848

**Published:** 2021-10-21

**Authors:** Michele Roccella, Luigi Vetri

**Affiliations:** 1 Department of Psychology, Educational Science and Human Movement, University of Palermo, 90128 Palermo, Italy; michele.roccella@unipa.it; 2 Oasi Research Institute-IRCCS, Via Conte Ruggero 73, 94018 Troina, Italy; 3 Clinic of Child and Adolescent Neuropsychiatry, Department of Mental Health and Physical and Preventive Medicine, University of Campania “Luigi Vanvitelli”, 81100 Caserta, Italy

Clinical psychology strives to fully grasp the person in his totality and in his individuality, helping him adequately address his own deep internal suffering and discomfort, social uneasiness, and harmonize his own needs, desires and, attachments. 

The practical effects of clinical psychology are to face adaptation problems and behaviour disorders in view of research, prevention, and psychological evaluation, and with the purpose of contributing to a proper psychotherapeutic intervention for the different forms of psychopathology.

From this perspective, this branch of psychology is strongly involved in processes of health promotion and in care systems, and the psychological dimension transversally permeates the entire healthcare activity.

Clinical psychology operates through the identification of risk and protective factors and contributes to the creation of treatment plans to promote the development and the balance of the person. To this end, in the various fields of intervention, clinical psychology concerns itself with ensuring that the clinical response of the care system takes into account both the physical and psychological distress of the individual and the family context, as well as the relational and social dimension, promoting an affective summary between a precision medicine approach and a global care scheme for the patient. 

Eating disorders (ED) are increasingly common conditions that are often overlooked, they are related to complex and damaging relationships with food and body image, and they are associated with serious health consequences. Studies about eating disorders represent an important field of research in clinical psychology.

Rogowska et al., in their study, aim to develop a new self-report questionnaire for the diagnosis of orthorexia nervosa. To this purpose, a total sample of 767 individuals were assessed by a 40-items questionnaire choosing to analyse a current review of the scientific literature. After a structural analysis, the number of items was reduced from 40 to 17 (TON-17), including three subscale factors (Control of food quality, Fixation of health and healthy diet, and Disorder symptoms) in a hierarchical, bi-factor structure. The study demonstrates that the TON-17 scale has good psychometric properties, stability, reliability, and construct validity, and therefore it could become a promising tool for assessing the risk of orthorexia nervosa [1].

The assessment of patients with neuropsychological disorders includes an evaluation of possible comorbidities that can increase the complexity of the disorder. Pruccoli investigates the impact of Autism Spectrum Disorder (ASD) traits, evaluated through the Autism Diagnostic Observation Schedule-Second Edition (ADOS-2) and the Autism-Spectrum Quotient (AQ), on the treatment intensity and outcomes in a group of adolescents hospitalised with Anorexia Nervosa. Their preliminary results evidenced that ASD traits were not significantly related to the treatment intensity or to the treatment outcomes; in fact, the treatment intensity and psychopathological outcomes were not different in patients with and without ASD diagnostic tests [2].

Mental pain (MP) is a subjective state of intense psychological distress related to several uncomfortable emotions, such as guilt, anguish, fear, panic, angst, loneliness, and helplessness, often associated with several psychopathological conditions, especially mood and anxiety disorders, personality disorders, and emotion dysregulation. Tomba et al., in their study, explore the presence of MP in patients with eating disorders, assessing 71 patients with MP and 90 matched controls. Patients with eating disorders show a significantly greater intensity and the most frequent cases of MP, and moreover MP is associated with disinhibited eating behavioural aspects and a depressive symptomatology, especially with suicidal tendencies, general and somatic anxiety, and insomnia. Therefore, the authors conclude that MP could become an important clinical marker for discriminating more severe cases of eating disorders [3].

Other factors influencing the phenomenology of eating disorders are irrational beliefs that are maladaptive cognitions about negative global evaluations of the self and others, awfulizing thoughts, low frustration tolerance beliefs, and demandingness. Tecuta et al., in their study, assessed 79 ED outpatients and 95 controls and demonstrated through a multivariate analysis of variance with post hoc comparisons that ED outpatients had greater awfulizing, more negative global evaluations, and a lower frustration tolerance than controls. The study provides experimental support for the hypothesis that feelings of inefficacy represent a core role in clinical models of EDs, and their evaluation is useful when assessing ED patients and planning cognitive–behavioral treatments [4].

Another relevant topic raised in the Special Issue is the role of major adverse events in psychological well-being. In their research, Krok et al. evaluated a sample of 225 healthcare workers, analysing the connection between stress, meaning making, the risk of contracting COVID-19, self-efficacy, meaning in life, and subjective well-being. A higher self-efficacy and meaning in life were linked to higher cognitive and affective dimensions of subjective well-being. On the contrary, a reduced risk of contracting COVID-19 was associated with a higher affective dimension. The authors’ results demonstrate that workers’ subjective well-being is highly related to the risk level of contracting COVID-19 and motivational factors, and therefore psychological interventions promoting adaptive forms of meaning making may be decisive in overcoming the anxiety over COVID-19 and improving the subjective well-being among healthcare personnel [5].

Similarly, Barone et al. evaluated the emotional concerns and psychological difficulties in a clinical sample of 40 young cancer survivors, assessing the self- and parent-reported emotional, somatic, and behavioral symptoms. Their data establish that a higher proportion of young survivors compared to controls had emotional distress and that the anxious symptom severity was significantly higher. The multi-informant assessments of the psychological profiles revealed that the survivors’ self-reports of depressive symptoms, somatic symptoms, and functional impairment were significantly related to the parent reports of child behavioral concerns, somatic complaints, and functional impairment [6].

San Martín–Valenzuela, in their interesting cross-sectional study on cognitive and motor differences in cirrhotic patients with and without minimal hepatic encephalopathy, evaluated gait, balance, hand strength motor speed, and cognitive performances in 76 patients with liver cirrhosis. Patients with minimal hepatic encephalopathy performed worse than patients without minimal hepatic encephalopathy both in motor performances (especially gait, centre of pressure movement, variability of hand strength performance, and hand motor speed) and in cognitive and autonomous functioning. Moreover, some motor variables are intimately related to cognitive aspects, and this association is absent in patients without minimal hepatic encephalopathy [7].

Ramiro-Cortijo et al., in their observational study, evaluated the impact of psychological health during pregnancy on maternal and neonatal outcomes. The authors, assessing 131 healthy pregnant women, found that maternal depression was associated with leukocytes, cholesterol, and pregnancy concerns, maternal resilience was associated with leukocytes and life satisfaction, and maternal optimism was associated with polyphenol levels and life satisfaction. Birth weight was associated with maternal resilience, red blood cells, and life satisfaction. The study results underlined the importance of considering preventive psychological health policies in the obstetric field [8].

Another field of interest is the impact of digital technologies on psychological well-being. Miceli et al. assessed 186 social networking site users in their cross-sectional study to verify if time perspective and attentional style could be related to social network addiction. Internal attentional style represents a key factor in controlling the association between high levels of time perspective and a high level of social network addiction. Social network-addicted users appear to be oriented toward internal stimuli like intrusive thoughts or feelings, and therefore social network addiction is similar to obsessive compulsive disorders, depression, or anxiety [9].

In conclusion, the Special Issue “Feature Papers in Clinical Psychology” represents an interesting collection of papers that may improve knowledge about several application areas of Clinical Psychology, providing some answers and opening several new questions that future studies will clarify. The need for a comprehensive psychological evaluation should be definitely considered as an essential element of any clinical assessment.
